# BSP Gene Silencing Inhibits Migration, Invasion, and Bone Metastasis of MDA-MB-231BO Human Breast Cancer Cells

**DOI:** 10.1371/journal.pone.0062936

**Published:** 2013-05-07

**Authors:** Jie Wang, Li Wang, Bing Xia, Chuanhong Yang, Huangwen Lai, Xiaodong Chen

**Affiliations:** 1 Department of Medical Research, Guangzhou General Hospital of Guangzhou Military Command, Guangzhou, Guangdong Province, China; 2 Department of Pathology, Guangzhou General Hospital of Guangzhou Military Command, Guangzhou, Guangdong Province, China; Thomas Jefferson University, United States of America

## Abstract

Bone sialoprotein (BSP) has been implicated in a variety of physiological and pathophysiological events, including tumor cell invasion, bone homing, adhesion, and matrix degradation. To explore the potential involvement of BSP in human breast cancer cell invasion and metastasis, we used retrovirus-mediated RNAi to deplete BSP levels in the human bone-seeking breast cancer cell line MDA-MB-231BO (231BO) and established the 231BO-BSP27 and 231BO-BSP81 cell clones. Cell proliferation, colony formation, wound healing, and the ability to invade into matrigel of these BSP-depleted clones were all decreased. Both 231BO-BSP27 cells and 231BO-BSP81 cells showed a significant (15.4% and 28.6% respectively) reduction of bone metastatic potential following intracardiac injection as determined by X-ray detection and by hematoxylin and eosin staining. Moreover, the expression of integrins αvβ3 and β3 was decreased in the BSP-silenced cells whereas ectopic BSP expression increased the integrins αvβ3 and β3 levels. These results together suggest that BSP silencing decreased the integrin αvβ3 and β3 levels, in turn inhibiting cell migration and invasion and decreasing the ability of the cells to metastasize to bone.

## Introduction

Breast cancer is a common malignancy among women. While locally contained in situ tumors can be surgically removed, the major threat arises from tumor cells that invade adjacent tissues or metastasize to distant sites [1]. Bone is a common site of metastasis; in fact, 64% of patients who die from breast cancer have bone metastases [2]. In addition, metastasis to the skeletal system is usually accompanied by osteolytic lesions, which can cause patients serious anguish. Unfortunately, bone metastases are usually insensitive to conventional breast cancer therapies.

Bone sialoprotein (BSP) is a highly phosphorylated and glycosylated secreted protein in the bone matrix, belonging to the class of molecules known as small integrin binding ligand N-linked glycoproteins, or SIBLINGs. Its expression is not restricted to bone, and, in fact, it has been described as a common extracellular protein secreted by human breast cancer cells [3]–[5]. BSP possesses a polyglutamate sequence that mediates binding to hydroxyapatite crystals [6], [7]. BSP also contains an integrin-binding RGD (Arg-Gly-Asp) sequence that may mediate protein binding to the cell surface [8] and may promote interactions between cells and the bone matrix through αvβ3 and αvβ5 integrin receptors [9]. Most (∼87%) breast carcinoma specimens show a significant elevation in BSP expression [3], and patients with preoperatively increased serum BSP levels are at high risk of subsequent bone metastases [10]. While over-expression of BSP promotes bone metastasis of human breast cancer cells in mouse models [5], [11], a decrease in BSP levels in breast cancer cells, using either antisense BSP cDNA or anti-BSP antibody, inhibits bone metastasis in vitro and in vivo [12]–[18].

Integrins are a family of transmembrane glycoproteins demonstrated to play a major role in tumor invasive and metastatic processes. Integrin αvβ3 is a key molecule that actively participates in tumor angiogenesis and metastasis [19]. Decreasing the levels of αv and β3 integrin subunits in cells can suppress cancer metastasis [20]. The anti-αv integrin monoclonal antibody intetumumab could bind cell surface proteins important for adhesion, invasion and angiogenesis in the metastatic cascade [21]. The binding of BSP and integrins could contribute to metastasis formation of breast cancer cells, and particularly bone metastasis. It has also been reported that αv integrin chain is markedly reduced in BSP(−/−) osteoclasts [22]. Expression of BSP in tumor cell lines could increase the levels of αv-containing integrins and the number of mature focal adhesions [23].

While the data obtained thus far are promising, limitations of previous studies, such as the immunogeneicity and the extracellular immunoreaction by the BSP antibody, and the temporary silencing of antisense oligonucleotides (ASOs) have made it challenging to define the exact role of BSP in bone metastasis. Small interfering RNAs (siRNA) can target mRNAs for degradation, and long-term stable reduction of BSP gene expression by siRNA may block BSP transcription and mimic gene mutations. Our previous study showed that silencing of BSP significantly inhibited the adhesion of MDA-MB-231BO cells (231BO for brevity) to bone matrix. Importantly, this adhesion may be a key component of BSP function in metastasis [24]. However, whether BSP-RNAi affects metastasis by blocking migration or invasion is unclear. Moreover, although it has been reported that BSP expression contributes to increased αv integrin, the effect of BSP silencing on integrin αvβ3 level is unclear. Here, we established two BSP gene-silenced cell clones of 231BO, termed 231BO-BSP27 and 231BO-BSP81, and showed that their proliferation, migration and invasion were suppressed. Following BSP silencing, the level of integrin αvβ3 was decreased in cell culture and the bone metastatic potential was dramatically decreased in nude mice. These data suggest that BSP silencing in human breast cancer cells could decrease the levels of integrins αvβ3 and β3, in turn could inhibit cell migration and invasion and decrease the ability to metastasize to bone.

## Materials and Methods

### Cell Lines, Reagents, and Antibodies

The bone-seeking human breast cancer cell line 231BO was generously provided by Dr. Toshiyuki Yoneda (University of Texas Health Science Center at San Antonio) [25]. Cells were cultured in DMEM (Hyclone) supplemented with 10% fetal bovine serum (FBS; Hyclone) at 37°C in a humidified atmosphere of 5% CO2. pSilencer™ 5.1 Retro Kit (Ambion, USA), T4 DNA ligase (NEB, USA), and Oligofectamine Reagent (Merck, USA) were used for shRNA construction. The β-actin (SANTA CRUZ, USA), GAPDH (CST, USA), BSP (SANTA CRUZ, USA), and integrin β3 (CST, USA) primary antibodies, the peroxidase-conjugated anti-mouse IgG (SANTA CRUZ, USA), and the ECL detection kit (Thermo, USA) were used in western blots. The integrin αvβ3 antibody clone LM609 (Merck Millipore, Germany), the FITC-conjugated anti-mouse IgG (CST, USA) and the PE-conjugated anti-mouse IgG (CST, USA) were used for flow cytometry. QuantiTect Rev Transcription kit (QIAGEN), QuantiTect SYBR Green PCR kit (QIAGEN), and Trizol (Invitrogen, UK) were used in quantitative RT-PCR. The primer sets used for PCR amplification of BSP and β-actin were synthesized by QIAGEN. MTT ([3-(4, 5-dimethylthiazol-2-yl)-2, 5-diphenyltetrazolium bromide], Sigma Aldrich, China), DMSO (Sigma Aldrich, China), and basement membrane matrigel (BD Biosciences, USA) were used in migration assays. Tartrate-resistant acid phosphatase (TRAP) staining was performed with a Sigma-Aldrich Acid Phosphatase kit. Elivision™ plus Polyer HRP (Mouse/Rabbit) IHC Kit (Maixin, China), DAB Detection Kit (Maixin, China) and the above-described antibodies were used for immunohistochemistry detection. pIRES2-EGFP vector was purchased from BD Biosciences Clonetech Inc, while the recombinant vector pIRES2-hBSP-EGFP was constructed by our lab.

### Establishment of Stable BSP-silenced Cell Lines

As described in the Table S1, the shRNA sequences (shBSP27, shBSP81, and shBSP100) were designed to target the 337–357th, the 774–794th and the 963–983 rd regions of the human BSP mRNA (NM_004967.3). Complementary oligonucleotides (sequences are shown in Table S1) were synthesized by Invitrogen and ligated into the pSilencer 5.1-U6 Retro vector using the T4 DNA ligase. *E. coli* Top10 cells were then transformed with the constructs and ampicillin-resistant colonies were selected. The inserts were confirmed by sequencing the plasmid using 5′-TTGTACACCCTAAGCCTC-3′ as the primer. The shRNA containing plasmids (1 µg/ml) were transfected into retroviral packaging 293 cells using Oligofectamine Reagent to propagate the retrovirus. pSilencer 5.1-U6 Retro Scrambled plasmid, which does not possess any significant homology to human, mouse, or rat gene sequences, served as a negative control. The culture medium containing the retrovirus of interest was then collected, and 231BO cells were infected. Cells were cultured for 2 weeks in the presence of 10 ng/ml puromycin to enrich the stable shRNA-expressing cells. Additional selections were performed every 3 months.

### Transfection of Recombinant pIRES2-hBSP-EGFP Plasmid

The 231BO-BSP27 cells were planted in 24-well plates with a density of 1.0×10^5^ cells/well the day before transfection. Lipofectamine™ 2000 (2 µL) was diluted in 50 µL serum-free DMEM, mixed gently and incubated for 5 min at room temperature. Plasmids (1 µg of pIRES2-EGFP or pIRES2-hBSP-EGFP) and Lipofectamine™ 2000 solution were added, followed by incubation for 20 min at room temperature to allow the formation of plasmid-Lipofectamine™ 2000 complexes. The complexes were added to 24-well plates in which the medium was changed with serum-free DMEM in advance, incubated for 10 h at 37°C in a CO_2_ incubator, and then cultured using DMEM with FBS to obtain cells for western bolt and flow cytometry analyses.

### Flow Cytometry Detection of Integrin αvβ3 Expression

Cells (1×10^6^) were washed twice and suspended with 1 mL PBS, followed by incubation with 5 µL LM609 αvβ3 antibody at 4°C for 45 min. After two washes with PBS, the cells were collected by low speed centrifugation (192 RCF, 5 min) and incubated with 2 µL FITC- or PE-conjugated goat anti-mouse IgG mAb at 4°C for 30 min in the dark, followed by two washes with PBS. The stained cells were then suspended in 500 µL PBS, and detected by MACSQuant Analyzer instruments (Miltenyi Biotec, Germany) and Guava easyCyte HT system (Merck Millipore, USA). The negative control was processed in the same way but without the anti-αvβ3 antibody.

### Western Blot Analysis

Cells were harvested and lysed with RIPA buffer containing the protease inhibitor PMSF. Equal amounts of protein (50 µg/lane) from the lysates were electrophoresed in PAGE and transferred to PVDF membranes. Membranes were blocked with 5% skim milk in TBS containing 0.1% Tween-20 for 2 h at room temperature and then incubated overnight at 4°C with primary antibody (1∶200 for anti-BSP; 1∶1000 for anti-integrin β3; 1∶2000 for anti-β-actin) in blocking solution. After incubation with horseradish peroxidase-conjugated anti-mouse IgG (1∶2000) in blocking solution for 60 min, the proteins were visualized with ECL detection kit.

### Quantitative RT-PCR

Total RNA was isolated from cells using Trizol (Thermo, USA). An aliquot (700 ng) of the RNA was used for qRT-PCR analysis with a QuantiTect Rev Transcription kit and a QuantiTect SYBR Green PCR kit. β-actin was used to normalize the protein loading. The conditions for the RT step were 42°C for 15 min and 95°C for 3 min. The thermal cycling conditions for PCR were 95°C for 15 min followed by 55 cycles at 94°C for 15 sec, 55°C for 30 sec, and 72°C for 30 sec per cycle.

### Cell Viability and Colony Formation Assays

Cells were plated in 96-well plates at a density of 2000 cells per well and incubated for 1, 2, 3, 4, 5, 6, or 7 days, followed by addition of MTT (20 µL, 5 mg/ml DMSO). After 4 h-incubation at 37°C, MTT residual was removed and 150 µL DMSO was added to dissolve the formazan crystals. Absorbance was measured at 570 nm to determine the cell viability. To determine colony formation, cells (1×10^3^) were cultred in 6-well plates for 2 weeks; this was followed by counting those colonies larger than 30 cells under an inverted microscope.

### Migration and Invasion Assays

Cells were plated in 6-well plates at a density of 5×10^5^ cells per well until they reached 90% confluence. A single wound was scraped with a pipette tip in the center of the cell monolayer, and the wells were washed with PBS to remove cell debris. After an additional 24 h of culture, wound healing was visualized with an inverted microscope. In vitro invasion was determined in 24-well transwell inserts with 8 µm-pore size filters (Corning) by following a method modified from the literature [26]. The filters were coated with 60 µL basement membrane matrigel diluted to 1∶3 with DMEM, air-dried and hydrated with 50 µL serum-free DMEM 30 min prior to cell addition. Cells were added to the upper chamber inserts at a concentration of 5×10^4^ cells in 0.2 ml of serum-free medium (at least 3 replicates for each sample). DMEM (500 µL) containing 20% FBS was added into the lower chamber. After incubation for 24 h at 37°C, cells in the upper part of the transwells were removed with a cotton swab, and the chambers were washed with PBS. Cells that had migrated were fixed with 4% paraformaldehyde and stained with 0.1% Crystal Violet for 15 min. Filters were photographed and dye elution using 500 µL 33% acetic acid solution was measured for absorbance at 570 nm to determine cell invasion.

### In vivo Metastasis Assays

Female athymic Balb/c nude mice (from Guangdong Medical Laboratory Animal Center), age-matched between 4 and 6 weeks, were housed in a pathogen free animal facility. Cells (2.5×10^5^ cells in 100 µL PBS) were injected into the left heart ventricle of nude mice as previously described [27]. Four weeks later, animals were anesthetized deeply and placed in the prone position. Whole body skeletons were X-rayed to detect the presence of bone lytic lesions, using a GE Digital Radiography instrument with X-ray exposure at 35 kV for 6 sec. All the brains, lungs, tibias and femurs were retained for histological study. After formalin-fixation, the bone tissues were transferred into a decalcification solution (4% EDTA, pH 7.2) for 4 weeks at 37°C and were embedded with paraffin. Other tissues were embedded without decalcification. Sections of 2–4 µm were stained with hematoxylin and eosin (HE) for histological analyses or were stained for tartrate-resistant acid phosphatase (TRAP) for osteoclast examination. Paraffin sections from the mouse bones were immunohistochemically (IHC) stained by incubation with the primary antibody (1∶100 for anti-BSP and 1∶200 for anti-integrin β3) for 14 h at 4°C, as instructed by the manual of the IHC Kit. After staining with a DAB kit, the sections were counterstained with haematoxylin. All the histological diagnoses and the quantifications of metastases were performed by two pathologists in a blinded manner.

### Ethics Statement

All animal experiments were carried out in a strict accordance with the recommendations in the Guide for the Management Ordinance of Laboratory Animals of Guangdong Province, China. The protocols were approved by the Committee of the Guangdong Laboratory Animals Monitoring Institute (Permit Number: SYXK(Jun)2007–033 and SYXK(Yue)2009–0100). All surgeries were performed under anesthesia, and all efforts were made to minimize suffering.

### Statistical Analysis

Densitometry of western blots was performed with Quantity One (Bio-Rad) and values were normalized to β-actin. RT-PCR was performed on a Rotor-Gene 6000 PCR detector and quantified using the cycle threshold (Ct) method and was normalized to β-actin. The wound healing rate was measured by comparing the area of the wound at 0 h and at 24 h.The number of bone, lung, and brain metastases and the TRAP-positive multinucleated cells in or around the metastatic lesions were assessed and photographed using an Image-Proa Express 6.0 software. The levels of integrin αvβ3 were analyzed using MACSQuantify (Miltenyi Biotec, Germany) and GuavaSoft 2.2 software (Merck Millipore, USA). All experiments were performed at least in triplicate. Data were presented as mean±SD (standard deviation). Statistical analysis was performed by one way ANOVA followed by the Bonferroni or Dunnett (2-sided) test for comparisons. The level of significance was set at p<0.05.

## Results

### BSP-silenced Cell Clones were Established

**Figure 1:**
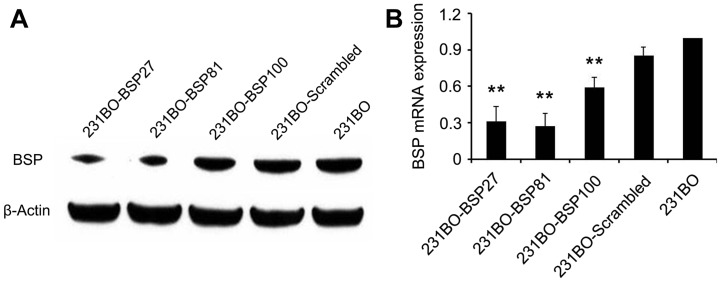
Confirmation of BSP knockdown detected by western blot (A) and qRT-PCR (B). The protein and mRNA levels of BSP are reduced significantly in both 231BO-BSP27 and 231BO-BSP81 cells. 231BO-BSP100 cells show a slight decrease, while 231BO-Scrambled cells show no significant difference from the parental 231BO cells. β-actin is used for control of loading. **p<0.001, significantly different from the 231BO.

As detected by western blot, the BSP27 or the BSP81 shRNA expressing clone of 231BO cells showed 69.3±4.1% or 75.2±5.2% reduction of the BSP protein, whereas the BSP100 shRNA clone and the scrambled clone only showed a 27.8±4.0% and an 8.8±2.5% decrease in BSP, respectively, when compared with the parental 231BO cells (Fig. 1A). Consistent with the reduction of the BSP protein, BSP mRNA in 231BO-BSP27, 231BO-BSP81, and 231BO-BSP100 cells were also decreased 69%, 73%, and 41% respectively, compared to 16% reduction in 231BO-Scrambled cells (Fig. 1B). We therefore used the 231BO-BSP27 and 231BO-BSP81 cells for further study on the effects of BSP on metastasis and other biological characteristics.

### Silencing BSP Inhibits Breast Cancer Cell Proliferation, Colony Formation, as well as Cell Migration and Invasion

**Figure 2:**
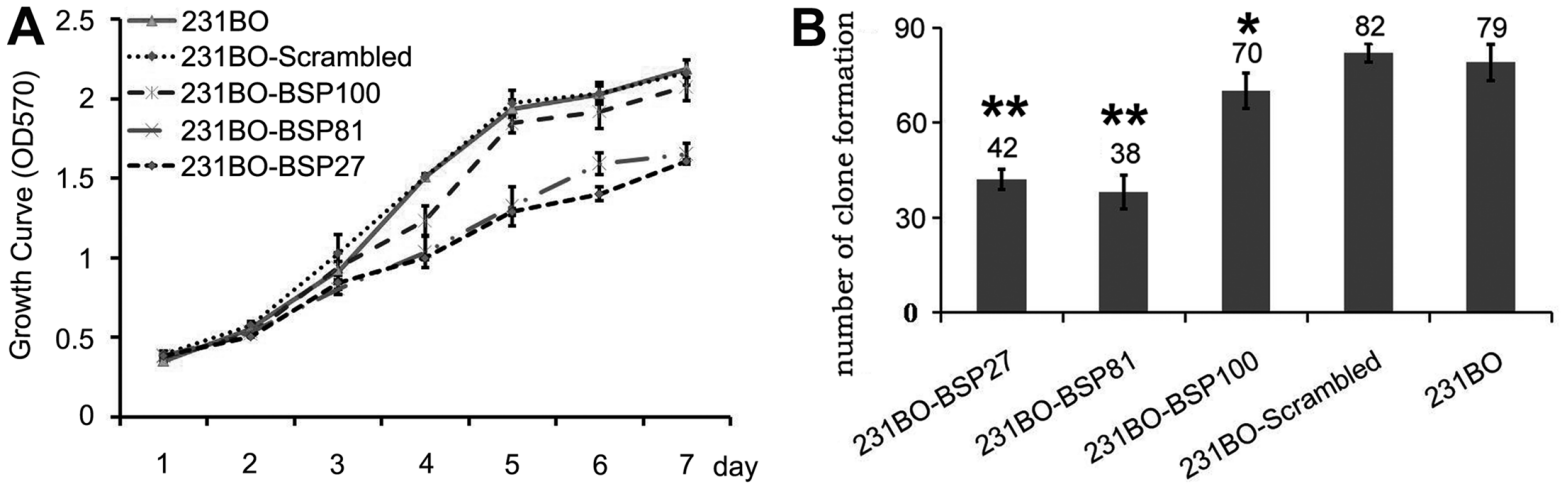
Silencing BSP inhibits 231BO cell growth and colony formation. (A) Growth curves of 231BO-BSP27, 231BO-BSP81, 231BO-BSP100, 231BO-Scrambled, and 231BO cells detected by MTT assay showing that the viability of 231BO-BSP27 and 231BO-BSP81 cells is significantly decreased from the fourth day on. (B). The numbers of colonies formed by the 231BO-BSP27 and 231BO-BSP81 cells are decreased at the endpoint of 2 weeks. *p<0.05 *vs* 231BO; **p<0.001 *vs* 231BO.

**Figure 3:**
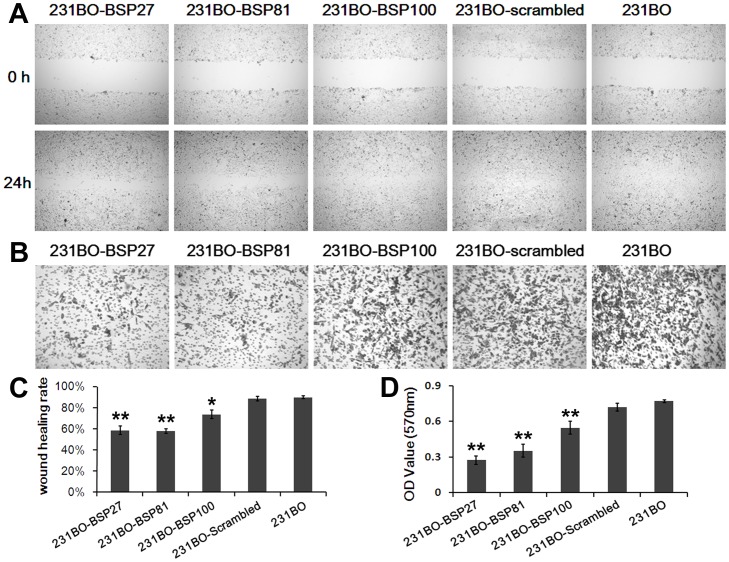
BSP silencing inhibits cell migration and invasion *in vitro*. (A) Wound healing assay for migration shows that non-BSP-silenced 231BO cells have almost completely invaded the wound whereas the BSP-silenced counterparts have only migrated poorly. (B) Transwell assay shows that BSP silencing reduces the invasion of 231BO cells through the matrigel barrier. (C) Wound healing rates of different cell clones. (D) OD_570_ values of the cells that have migrated through the transwell after 24 h show that the invasion abilities of the 231BO-BSP27 cells and the 231BO-BSP81 cells are decreased significantly compared with the 231BO-Scrambled cells. *p<0.05 *vs* 231BO; **p<0.001 *vs* 231BO.

MTT assay showed that the viability of BSP-silenced cells was not changed during the first 3 days but was slightly decreased at the 4^th^ day (p<0.01), compared to the cells with higher BSP levels (Fig. 2A). Colony formation showed a 48.8% or a 53.7% decrease in 231BO-BSP27 or 231BO-BSP81 cells compared to 231BO-Scrambled cells (p<0.05) (Fig. 2B). One day after wound healing, the 231BO and 231BO-Scrambled cells almost completely invaded the wound, whereas the 231BO-BSP27 and the 23BO-BSP81 cells migrated only poorly, showing a decrease of 34.8% and 35.7% in wound healing (Fig. 3A and 3C). In the transwell assay for invasion, BSP silencing rescued the cell invasion through the matrigel barrier, as the invasion ability of 231BO-BSP27 cells decreased 61.9% compared to 231BO-scrambled cells and 231BO-BSP81 cells decreased 50.9% (p<0.05) (Fig. 3B and 3D).

### Silencing BSP Inhibits Breast Cancer Metastasis in vivo

**Figure 4:**
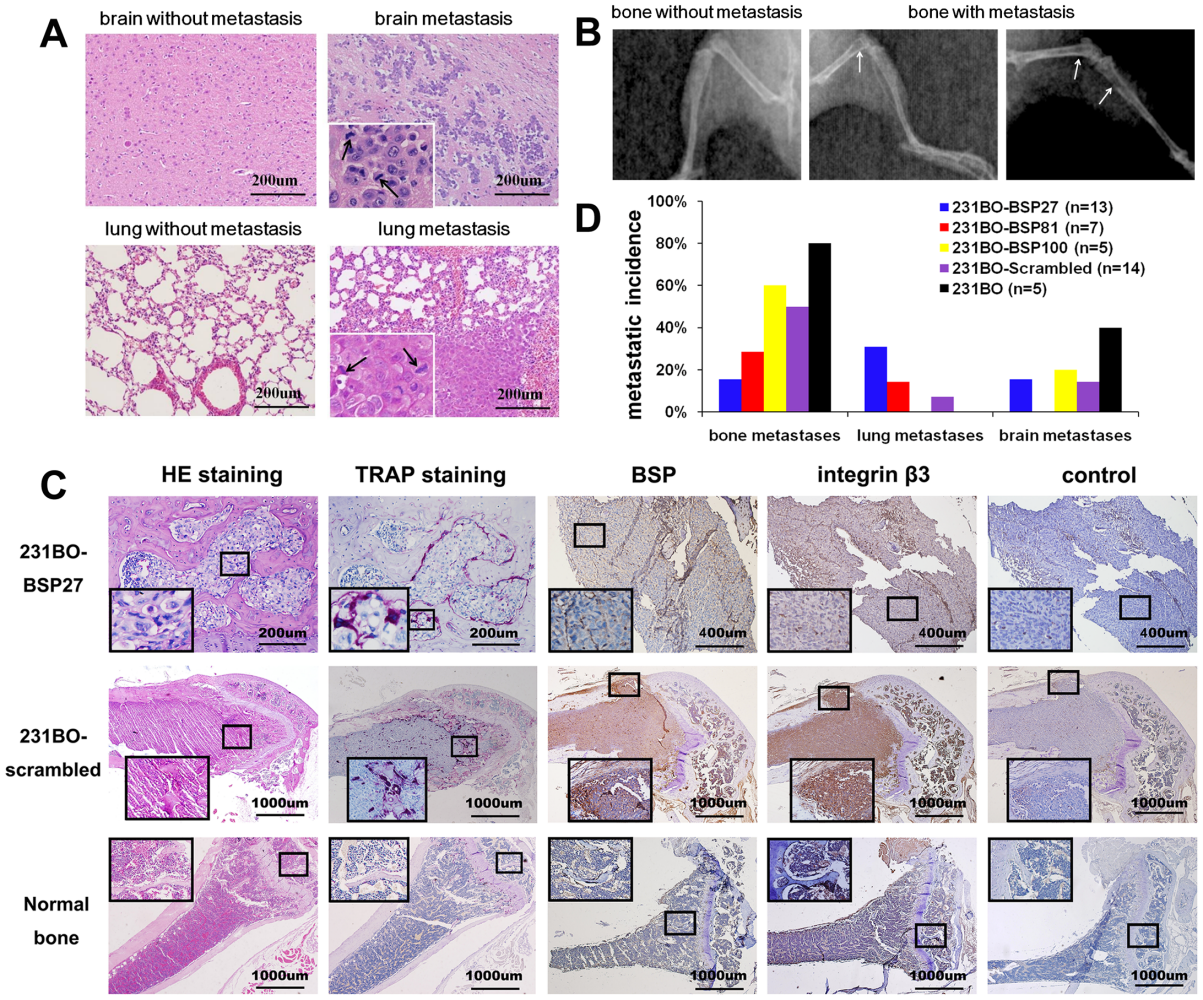
Metastasis in nude mice. (A) HE staining of brain and lung tissues. Arrows point to pathological mitotic figure. (B) X-ray scanning of bone lesions in nude mice. (C) HE staining, TRAP staining and immunohistochemical staining of tibias and femurs. (D) Metastatic incidences in the bone, lung and brain, as the percentage of the nude mice that show the lesion. “n” indicates the number of nude mice in each group.

Four weeks after intracardiac injection of the BSP-silenced cells, metastases to bone, brain, and lung were observed on HE-stained sections in both BSP-silenced and non-silenced groups (Fig. 4A and 4D), and metastasis-caused osteolytic lesions of bone were detected by X-ray (Fig. 4B) with active osteoclasts confirmed by TRAP staining (Fig. 4C). The BSP and integrin β3 proteins in bone metastasis were detected by immunohistochemical staining. Because of the limited number of brain and lung metastases, we could not determine whether the small difference was only due to the BSP silencing. Since X-ray only showed the bone mineral density and HE staining only showed histology, lesion size could not be quantified accurately. Moreover, bone metastasis is a dynamic process and the lesion size changes with time. Therefore, we only quantified the number of mice with metastases (Fig. 4D), which is much more reliable than other parameters. The incidence of bone metastasis was only 15.38% in 13 mice belonging to the 231BO-BSP27 group and 28.57% in 7 mice of the 231BO-BSP81 group. In contrast, the incidence of metastasis was 60% in 5 mice of the 231BO-BSP100 group and 50% in 14 mice of the 231BO-scrambled group. It was 80% in 5 mice belonging to the 231BO group.

Bone sections containing metastases of the 231BO-BSP27 and the 231BO-BSP81 cells showed significantly low levels of BSP and integrin β3 compared with the bones from the 231BO-Scrambled and 231BO cells (Fig. 4C). In all 5 groups, the lesion sizes were different among individuals within the same group. The ability of cancer cells to stimulate osteoclast formation is an important requirement for the establishment and development of osteolytic metastases [28]. We observed many osteoclasts in bone metastatic regions, especially between the bone matrix and the metastatic lesions (Fig. 4C). As shown in the figures of TRAP staining, many osteoclasts joined together, so we could not get the exact number in a metastatic. Moreover, a big bone lesion may not have a high density of osteoclasts, which may be relevant to the interaction between metastatic tumor cells and bone marrow cells. When counting the approximate number of osteoclasts under a microscope, we did not find any significant differences between these groups, suggesting that BSP expression may not obviously influence this cell type.

### The Expression of Integrin β3 and αvβ3 Related Closely to BSP Level

**Figure 5:**
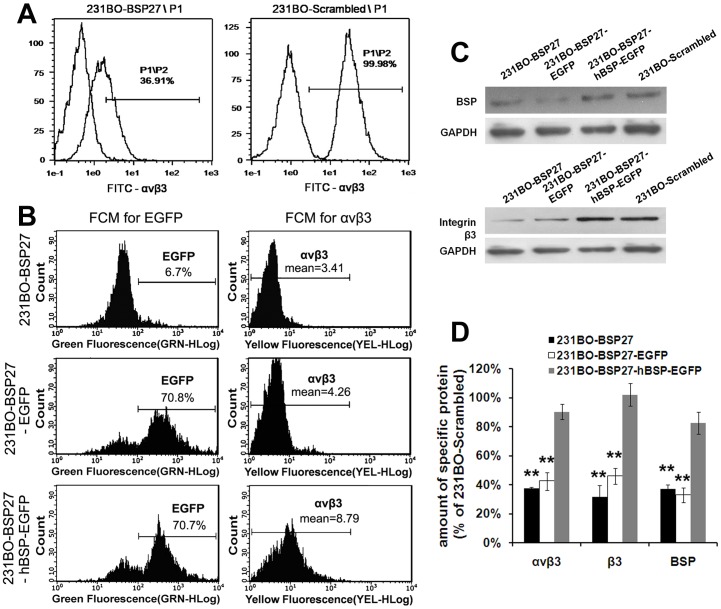
The levels of BSP and integrins. (A) Flow cytometry (FCM) analyses of αvβ3 protein in the 231BO-BSP27 and 23BO-Scrambled cells, with their negative control in front. The αvβ3 level is lower in the 231-BSP27 cells than in the 231BO-Scrambled cells. (B) FCM analyses for EGFP are used to detect the percentage of the pIRES2-EGFP and pIRES2-hBSP-EGFP transfected cells, while FCM analyses for αvβ3 detect the effect of BSP on the αvβ3 expression. The αvβ3 level is decreased in BSP-depleted cells but is restored by transfection with the pIRES2-hBSP-EGFP. (C) Western blots show that the β3 level is decreased in BSP-depleted cells but is restored by BSP re-expression, with GAPDH as the loading control. (D) Comparisons of the αvβ3, β3 and BSP levels in four cell clones. **p<0.001 *vs* 231BO-Scrambled.

Flow cytometry results showed that integrin αvβ3 was expressed at a higher level in 231BO-Scrambled cells but at a lower level in 23BO-BSP27 cells (Fig. 5A). Wondering whether αvβ3 might play a role in BSP-induced breast cancer metastasis, we transfected the 231BO-BSP27 cells with a pIRES2-hBSP-EGFP construct or the pIRES2-EGFP control construct, followed by detection of the BSP, β3 and αvβ3 levels with western blot and flow cytometry. As expected, the αvβ3 (Fig. 5B) and β3 (Fig. 5C) levels were lower in the 231BO-BSP27 and 231BO-BSP27-EGFP cells than in the 231BO-BSP27-hBSP-EGFP and 231BO-Scrambled cells. Particularly notable was that the levels of β3 and αvβ3 were changed with the same trend as the BSP level in the cells (Fig. 5D).

## Discussion

More than 50% of women that are diagnosed with breast cancer develop bone metastases with the risk of skeletal-related events. As a result, their quality of life is severely reduced [29]. As a member of the SIBLING gene family, BSP represents a potential target for the inhibition of cancer cell migration, invasion, bone homing, and progression to metastasis [30]. Previous studies have documented the ability to down-regulate BSP expression by ASOs [12], [13], [18]. In these studies, the maximum inhibition of BSP protein expression reached 75%. Their results showed an inhibition of proliferation, colony formation, and migration of MDA-MB-231 breast cancer cells. Moreover, antisense oligonucleotides of BSP could inhibit formation of osteolytic metastasis in nude rats near 50%, and also significantly reduced metastatic lesions. Use of a BSP antibody [14]–[17] could also reduce cell migration, invasion, and the generation of osteolytic lesions caused by human breast cancer cells in nude rat models. Compared to untreated controls, the size of lytic lesions in bone was significantly reduced, and approximately 20% of the rats did not suffer from discernible lytic lesions following BSP antibody treatment [15].

The potencies of cancer cells to adhere to bone matrix and to promote osteoclast formation are key requirements for the establishment and growth of bone metastases [28]. Our previous study had shown that silencing of BSP significantly inhibited the adhesion of 231BO cells to bone matrix and, thus, inhibited metastasis [24]. Seemingly contradictory, exogenously added BSP peptides appeared to inhibit the bone homing preference of breast cancer cells [31]. This may be due to the fact that BSP-peptide analogues may not completely represent endogenous BSP. Most notably, the BSP-peptide analogues did not contain the polyglutamate sequence that is involved in BSP binding to hydroxyapatite crystals. Thus, one possibility is that the BSP-peptide analogues may bind to cancer cells but not to the bone matrix. In contrast, endogenous BSP, containing the polyglutamate sequence, may promote interactions between cancer cells and the bone matrix.

In this study, two recombinant retroviruses targeting the BSP gene were generated and stably transferred into a bone-seeking breast cancer cell line 231BO. Although expression of BSP in 231BO-BSP27 cells and 231BO-BSP81 cells remained at 30.7% and 24.8% of that in the control cells, the reduction in BSP expression caused profound changes in metastatic characteristics. Our results strongly suggest that cell proliferation, colony formation, and migration of breast cancer cells are decreased in BSP-silenced cells compared with control cells. In fact, BSP shows a great potential to play roles in each critical step of the metastasis. All our data support earlier studies that suggest a critical role for BSP in breast cancer metastasis. Most importantly, BSP silencing significantly inhibits bone metastasis of breast cancer cells in nude mice. During the progression to osteolytic metastases, osteoclasts are active and play an important role in bone lesion formation. Breast cancer cells in the bone microenvironment can promote osteoclast formation and activity through factors such as PTHrP and IL11. Additionally, bone marrow stromal cells can secrete TGF-β_1_, RANKL, and other factors to accelerate tumor growth [32]. In bone metastasis slides, we observe many osteoclasts, especially at the edge of the bone metastases. However, by counting the number of osteoclasts, we do not detect statistically significant differences among these groups, suggesting that BSP silencing has no obvious influence on the activity of osteoclasts in the bone metastatic site. These in vivo studies show a high bone metastatic incidence but relatively low metastatic incidences to the brain and lung. Thus, although lung metastases in BSP silencing groups are slightly higher than those in non-silencing groups, we are unable to determine whether BSP can influence lung metastasis because of the small number of occurrence. The same is true of brain metastasis. Addressing these questions in the future will require establishment of new models with higher metastatic incidence to these organs.

BSP is recognized as an important ligand for αvβ3, an integrin that may confer bone metastatic potential to tumor cells [33]. We found that expression of this integrin was positively correlated with BSP levels in metastatic breast cancer cells. It has been shown previously that BSP expression in tumor cells increases the levels of αv-containing integrins [34]. However, such regulation at the protein level of integrin β3 has not yet been shown. Moreover, we show that the β3 and αvβ3 levels are positively correlated with the level of BSP. Thus, the regulation by BSP on integrin may imply an important mechanism of how BSP influences bone metastasis, which encourages us to further investigate the mechanism of αvβ3 in the role of BSP-related breast cancer metastasis.

In conclusion, both *in vitro* and *in vivo* studies support a role for BSP in bone metastasis. Inhibition of the expression of integrin β3 and αvβ3 by BSP may be an underlying mechanism, although more mechanistic insight of this regulation is still obscure. Moreover, the roles of BSP and of these integrins in breast cancer metastasis to the lung and brain are other interesting areas of research. Our study also suggests a new strategy to use BSP siRNA for prevention or adjuvant therapy of breast cancer.

## Supporting Information

Table S1 **The shRNA sequences targeting human BSP mRNA** (target sequences and their complementary sequences are underlined).
(DOC)

## References

[1] Talmadge JE, Fidler IJ (2010) AACR centennial series: the biology of cancer metastasis: historical perspective. Cancer Res 70(14): 5649–5669. 20610625 10.1158/0008-5472.CAN-10-1040 PMC4037932

[2] Yoneda T (1998) Cellular and molecular mechanisms of breast and prostate cancer metastasis to bone. Eur J Cancer 34(2): 240–245. 9741327 10.1016/s0959-8049(97)10132-0

[3] Bellahcène A, Merville MP, Castronovo V (1994) Expression of bone sialoprotein, a bone matrix protein, in human breast cancer. Cancer Res 54(11): 2823–2826. 8187059

[4] Bellahcene A, Kroll M, Liebens F, Castronovo V (1996) Bone sialoprotein expression in primary human breast cancer is associated with bone metastases development. J Bone Miner Res 11(5): 665–670. 9157781 10.1002/jbmr.5650110514

[5] Tu Q, Zhang J, Fix A, Brewer E, Li YP, et al (2009) Targeted overexpression of BSP in osteoclasts promotes bone metastasis of breast cancer cells. J Cell Physiol 218(1): 135–145. 18756497 10.1002/jcp.21576 PMC2666312

[6] Harris NL, Rattray KR, Tye CE, Underhill TM, Somerman MJ, et al (2000) Functional analysis of bone sialoprotein: identification of the hydroxyapatite-nucleating and cell-binding domains by recombinant peptide expression and site-directed mutagenesis. Bone 27(6): 795–802. 11113390 10.1016/s8756-3282(00)00392-6

[7] Goldberg HA, Warner KJ, Li MC, Hunter GK (2001) Binding of bone sialoprotein, osteopontin and synthetic polypeptides to hydroxyapatite. Connect Tissue Res 42(1): 25–37. 11696986 10.3109/03008200109014246

[8] Wuttke M, Müller S, Nitsche DP, Paulsson M, Hanisch FG, et al (2001) Structural characterization of human recombinant and bone-derived bone sialoprotein. Functional implications for cell attachment and hydroxyapatite binding. J Biol Chem 276(39): 36839–36848. 11459848 10.1074/jbc.M105689200

[9] Sung V, Stubbs JT 3rd, Fisher L, Aaron AD, Thompson EW, *et al* (1998) Bone sialoprotein supports breast cancer cell adhesion proliferation and migration through differential usage of the alpha(v)beta3 and alpha(v)beta5 integrins. J Cell Physiol 176(3): 482–94. 9699501 10.1002/(SICI)1097-4652(199809)176:3<482::AID-JCP5>3.0.CO;2-K

[10] Diel IJ, Solomayer EF, Seibel MJ, Pfeilschifter J, Maisenbacher H, et al (1999) Serum bone sialoprotein in patients with primary breast cancer is a prognostic marker for subsequent bone metastasis. Clin Cancer Res 5(12): 3914–3919. 10632320

[11] Zhang JH, Tang J, Wang J, Ma W, Zheng W, et al (2003) Over-expression of bone sialoprotein enhances bone metastasis of human breast cancer cells in a mouse model. Int J Oncol 23(4): 1043–8. 12963984

[12] Adwan H, Bäuerle T, Najajreh Y, et al (2004) Decreased levels of osteopontin and bone sialoprotein II are correlated with reduced proliferation, colony formation, and migration of GFP-MDA-MB-231 cells. Int J Oncol 24(5): 1235–1244. 15067347

[13] Adwan H, Bäuerle TJ, Berger MR (2004) Downregulation of osteopontin and bone sialoprotein II is related to reduced colony formation and metastasis formation of MDA-MB-231 human breast cancer cells. Cancer Gene Ther 11(2): 109–120. 14647232 10.1038/sj.cgt.7700659

[14] Cogan G, Bansal AK, Ibrahim S, Zhu B, Goldberg HA, et al (2004) Analysis of human bone sialoprotein in normal and pathological tissues using a monoclonal antibody (BSP 1.2 mab). Connect Tissue Res 45(1): 60–71. 15203941 10.1080/03008200490278151

[15] Bäuerle T, Adwan H, Kiessling F, Hilbig H, Armbruster FP, et al (2005) Characterization of a rat model with site-specific bone metastasis induced by MDA-MB-231 breast cancer cells and its application to the effects of an antibody against bone sialoprotein. Int J Cancer 115(2): 177–186. 15688393 10.1002/ijc.20840

[16] Bäuerle T, Peterschmitt J, Hilbig H, Kiessling F, Armbruster FP, et al (2006) Treatment of bone metastasis induced by MDA-MB-231 breast cancer cells with an antibody against bone sialoprotein. Int J Oncol 28(3): 573–583. 16465361

[17] Peterschmitt J, Bäuerle T, Berger MR (2007) Effect of zoledronic acid and an antibody against bone sialoprotein II on MDA-MB-231(GFP) breast cancer cells in vitro and on osteolytic lesions induced in vivo by this cell line in nude rats. Clin Exp Metastasis 24(6): 449–459. 17636409 10.1007/s10585-007-9082-x

[18] Elazar V, Adwan H, Bäuerle T, Rohekar K, Golomb G, et al (2010) Sustained delivery and efficacy of polymeric nanoparticles containing osteopontin and bone sialoprotein antisenses in rats with breast cancer bone metastasis. Int J Cancer 126(7): 1749–1760. 19739076 10.1002/ijc.24890

[19] Liu D, Wang C, Li C, Zhang X, Zhang B, et al (2011) Production and characterization of a humanized single-chain antibody against human integrin alphav beta3 protein. J Biol Chem. 286(27): 24500–7. 10.1074/jbc.M110.211847 PMC3129229 21606501

[20] Liu H, Radisky DC, Yang D, Xu R, Radisky ES, et al (2012) MYC suppresses cancer metastasis by direct transcriptional silencing of αv and β3 integrin subunits. Nat Cell Biol. 14(6): 567–74. 10.1038/ncb2491 PMC3366024 22581054

[21] Wu YJ, Muldoon LL, Gahramanov S, Kraemer DF, Marshall DJ, et al (2012) Targeting α(V)-integrins decreased metastasis and increased survival in a nude rat breast cancerbrain metastasis model. J Neurooncol 110(1): 27–36. 22842979 10.1007/s11060-012-0942-0 PMC3726254

[22] Boudiffa M, Wade-Gueye NM, Guignandon A, Vanden-Bossche A, Sabido O, et al (2010) Bone sialoprotein deficiency impairs osteoclastogenesis and mineral resorption in vitro. J Bone Miner Res 25(12): 2669–79. 20812227 10.1002/jbmr.245

[23] Gordon JA, Sodek J, Hunter GK, Goldberg HA (2009) Bone sialoprotein stimulates focal adhesion-related signaling pathways: role in migration and survival of breast and prostate cancer cells. J Cell Biochem 107(6): 1118–28. 19492334 10.1002/jcb.22211

[24] Wang L, Wang J, Yang D, Yang C, Xia B, et al (2011) Inhibitory effect of bone sialoprotein silencing on the adhesion ability of breast cancer cells to bone matrix. Sheng Wu Gong Cheng Xue Bao 27(2): 233–239. 21650048

[25] Yoneda T, Williams PJ, Hiraga T, Niewolna M, Nishimura R, et al (2001) A bone-seeking clone exhibits different biological properties from the MDA-MB-231 parental human breast cancer cells and a brain-seeking clone in vivo and in vitro. J Bone Miner Res 16(8): 1486–95. 11499871 10.1359/jbmr.2001.16.8.1486

[26] Adorno M, Cordenonsi M, Montagner M, Dupont S, Wong C, et al (2009) A Mutant-p53/Smad complex opposes p63 to empower TGFbeta-induced metastasis. Cell 137(1): 87–98. 19345189 10.1016/j.cell.2009.01.039

[27] Guise TA, Yin JJ, Taylor SD, Kumagai Y, Dallas M, et al (1996) Evidence for a causal role of parathyroid hormone-related protein in the pathogenesis of human breast cancer-mediated osteolysis. J Clin Invest 98(7): 1544–9. 8833902 10.1172/JCI118947 PMC507586

[28] Sterling JA, Edwards JR, Martin TJ, Mundy GR (2011) Advances in the biology of bone metastasis: how the skeleton affects tumor behavior. Bone 48(1): 6–15. 20643235 10.1016/j.bone.2010.07.015

[29] Jensen AØ, Jacobsen JB, Nørgaard M, Yong M, Fryzek JP, et al (2011) Incidence of bone metastases and skeletal-related events in breast cancer patients: a population-based cohort study in Denmark. BMC Cancer 11: 29. 21261987 10.1186/1471-2407-11-29 PMC3037922

[30] Bellahcène A, Castronovo V, Ogbureke KU, Fisher LW, Fedarko NS, et al (2008) Small integrin-binding ligand N-linked glycoproteins (SIBLINGs): multifunctional proteins in cancer. Nat Rev Cancer 8(3): 212–26. 18292776 10.1038/nrc2345 PMC2484121

[31] Byzova TV, Kim W, Midura RJ, Plow EF (2000) Activation of integrin alpha(V)beta(3) regulates cell adhesion and migration to bone Sialoprotein. Exp Cell Res 254(2): 299–308. 10640428 10.1006/excr.1999.4765

[32] Onishi T, Hayashi N, Theriault RL, Hortobagyi GN, Ueno NT (2010) Future directions of bone-targeted therapy for metastatic breast cancer. Nat Rev Clin Oncol 7(11): 641–651. 20808302 10.1038/nrclinonc.2010.134

[33] Pécheur I, Peyruchaud O, Serre CM, Guglielmi J, Voland C, et al (2002) Integrin alpha(v)beta3 expression confers on tumor cells a greater propensity to metastasize to bone. FASEB J 16(10): 1266–8. 12153995 10.1096/fj.01-0911fje

[34] Gordon JA, Sodek J, Hunter GK, Goldberg HA (2009) Bone sialoprotein stimulates focal adhesion-related signaling pathways: role in migration and survival of breast and prostate cancer cells. J Cell Biochem 107(6): 1118–28. 19492334 10.1002/jcb.22211

