# Correction: BSP Gene Silencing Inhibits Migration, Invasion, and Bone Metastasis of MDA-MB-231BO Human Breast Cancer Cells

**DOI:** 10.1371/annotation/d514b852-6b48-4720-a245-080daeb5eb05

**Published:** 2014-01-06

**Authors:** Jie Wang, Li Wang, Bing Xia, Chuanhong Yang, Huangwen Lai, Xiaodong Chen

There is an error in the reference list. The correct source for Reference 31 is: 

Van der Pluijm G, Vloedgraven HJ, Ivanov B, Robey FA, Grzesik WJ, et al. (1996). Bone sialoprotein peptides are potent inhibitors of breast cancer cell adhesion to bone. Cancer research,56(8), 1948-1955. 

