# Supplementary material for: BSP Gene Silencing Inhibits Migration, Invasion, and Bone Metastasis of MDA-MB-231BO Human Breast Cancer Cells
Source: PLoS One. 2013 May 7;8(5):e62936. doi: 10.1371/journal.pone.0062936 (PMC3647072; doi:10.1371/journal.pone.0062936)
Supplement: Table S1 — The shRNA sequences targeting human BSP mRNA (target sequences and their complementary sequences are underlined). (DOC) [file pone.0062936.s001.doc]

**Table S1: The shRNA sequences targeting human BSP mRNA** (target sequences and their complementary sequences are underlined):

| shBSP27 positive-sense: | 5'-GATCCGAAGAGGAGACTTCAAATGTTCAAGAGACATTTGAAGTCTCCTCTTCTTTTTTGGAAA-3' |
| --- | --- |
| shBSP27 antisense: | 5'-AGCTTTTCCAAAAAAGAAGAGGAGACTTCAAATGTCTCTTGAA CATTTGAAGTCTCCTCTTCG-3' |
| shBSP81 positive-sense: | 5'-GATCCGACAACAACCTCTCCAAATTTCAAGAGAATTTGGAGAGGTTGTTGTCTTTTTTGGAAA-3' |
| shBSP81 antisense: | 5'-AGCTTTTCCAAAAAAGACAACAACCTCTCCAAATTCTCTTGAAATTTGGAGAGGTTGTTGTCG-3' |
| shBSP100 positive-sense: | 5'-GATCCGTTACCGAGCCTATGAAGATTTCAAGAGAATCTTCATAGGCTCGGTAATTTTTTGGAAA-3' |
| shBSP100 antisense: | 5'-AGCTTTTCCAAAAAATTACCGAGCCTATGAAGATTCTCTTGAAATCTTCATAGGCTCGGTAACG-3' |
